# Supplementary material for: Experimental Investigation of the Novel Periodic Feed Pressure Technique in Minimizing Fouling during the Filtration of Oily Water Systems Using Ceramic Membranes
Source: Membranes (Basel). 2022 Sep 8;12(9):868. doi: 10.3390/membranes12090868 (PMC9504730; doi:10.3390/membranes12090868)
Supplement: Supplementary file 1 [file membranes-12-00868-s001.zip › membranes-1907038-supplementary.pdf]

membranes

MDPI

Supplementary Materials

Experimental investigation of the novel periodic feed pressure technique in minimizing fouling during the filtration of oily water systems using ceramic membranes

Mohamed Echakouri, Amgad Salama* and Amr Henni

Process System Engineering, Produced Water Treatment Laboratory, Faculty of Engineering and Applied Science, University of Regina, Regina, SK S4S 0A2, Canada

* Correspondence: amgad.salama@uregina.ca

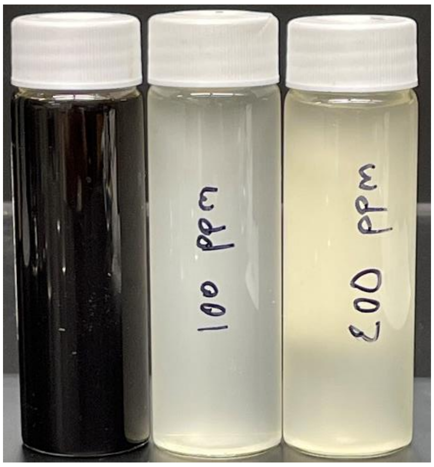

Figure S1. From left to right, Bakken oil, feed 100ppm, and feed 200ppm.

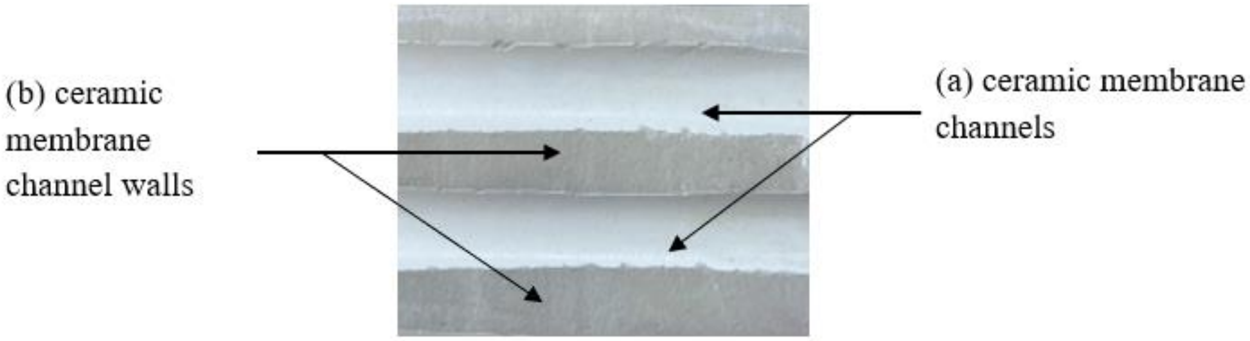

Figure S2. New ceramic membrane: (**a**) Ceramic membrane channels, (**b**) ceramic membrane channel walls.

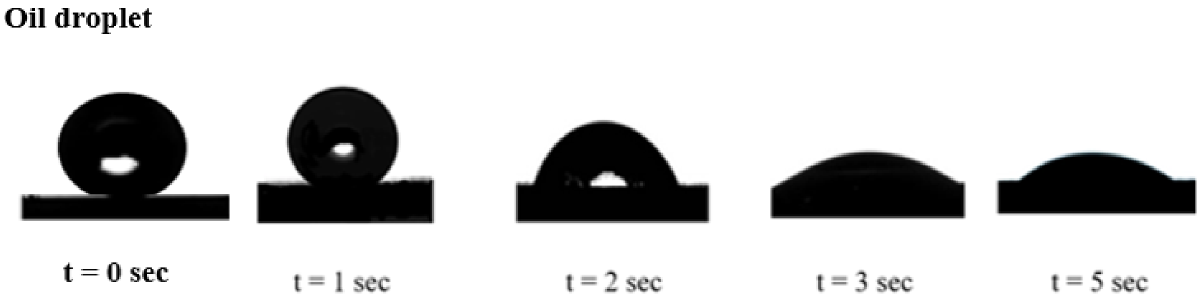

Figure S3. Sequential Images of the contact angle of a Bakken oil droplet at the ceramic membrane surface.

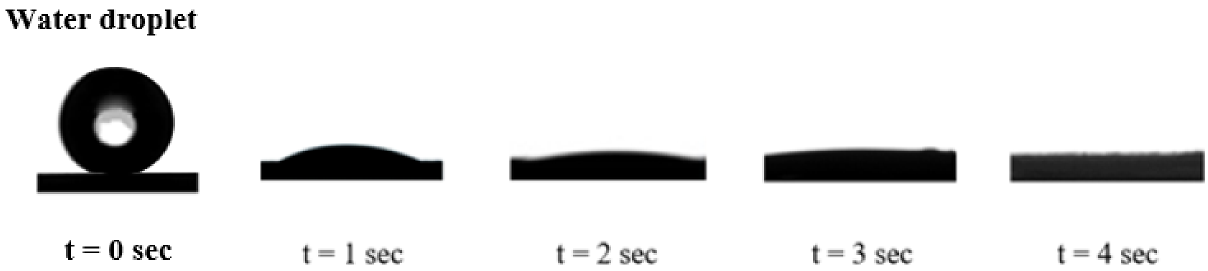

Figure S4. Successive Images of the contact angle of a water droplet at the ceramic membrane surface.

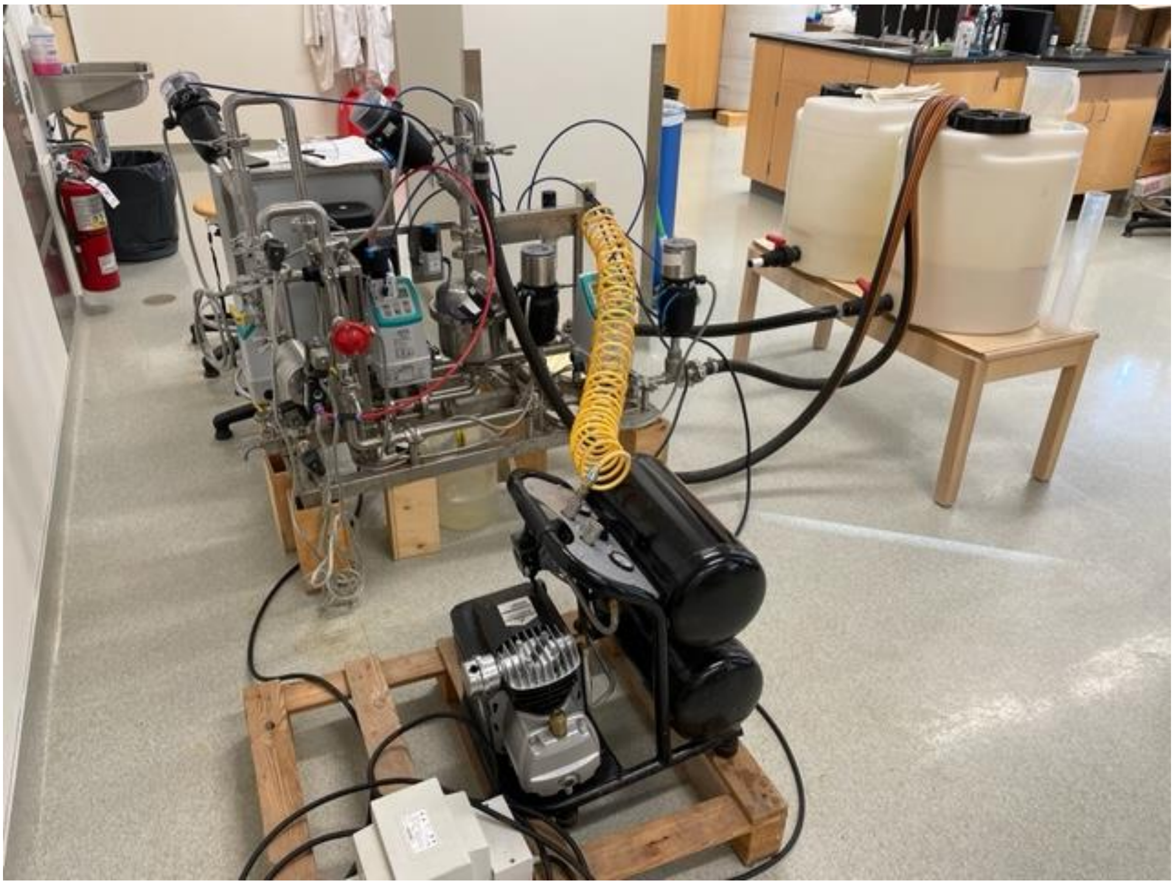

Figure S5. LabBrain ceramic membrane filtration unit.

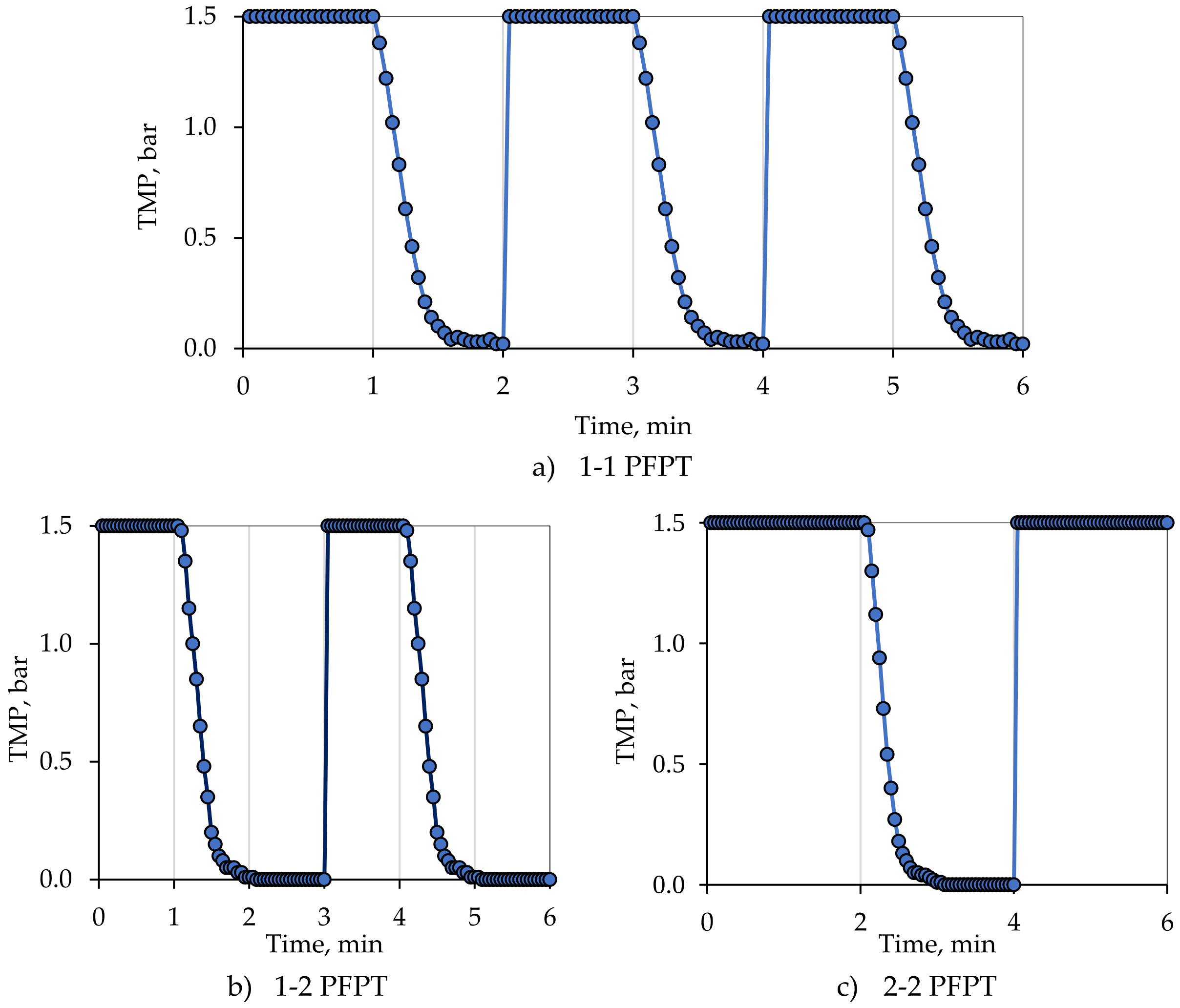

Figure S6. A: Different pressure patterns of the PFPT. (**a**) 1-1 PFPT, (**b**) 1-2 PFPT, and (**c**) 2-2 PFPT.

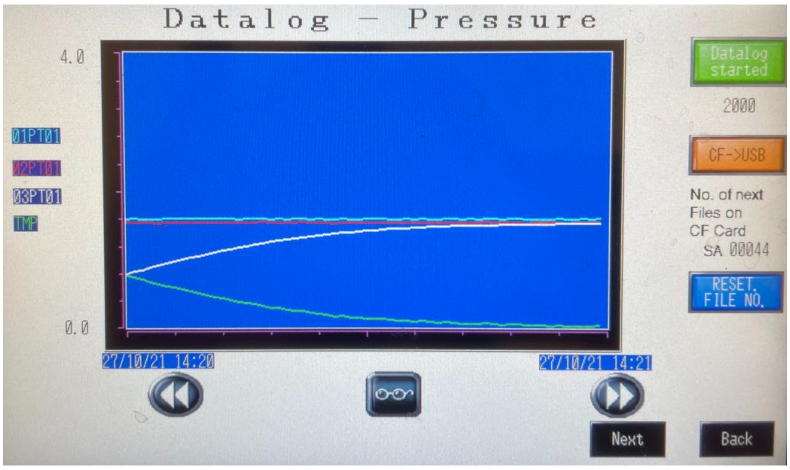

Figure S7. Control panel in the LabBrain filtration unit and Data log Pressure during the cleaning cycle (TMP (green line) tends to 0 bar).

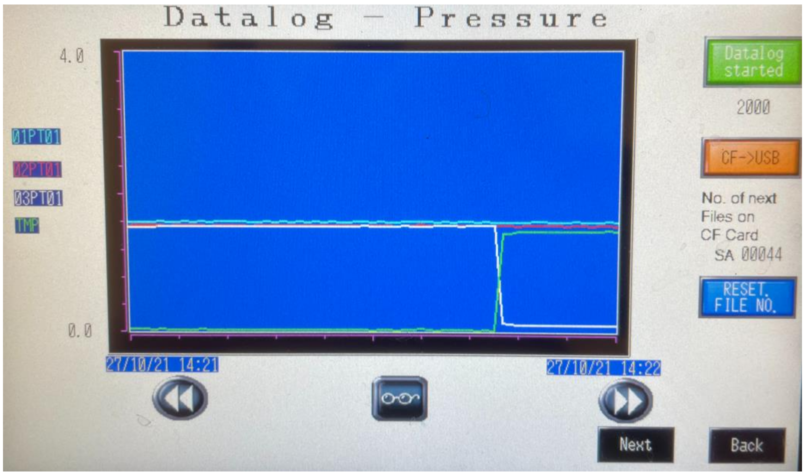

Figure S8. Control panel in the LabBrain filtration unit and Data log Pressure during the PFPT Filtration cycle (TMP (green line) =1.5 bar).

	Internal channel ceramic membrane		
Feed 100ppm	At, t = 0 min **new ceramic membrane is used for the experiment**	At, t = 120 min **Fouled membrane At the end of the experiment**	**Water post-cleaned Ceramic membrane**
No PFPT	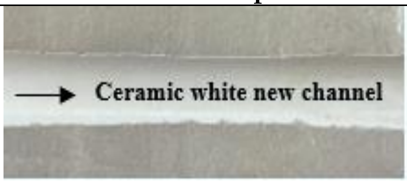	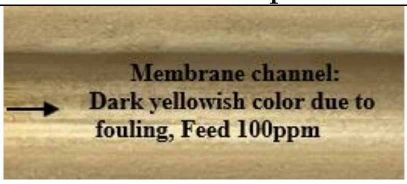	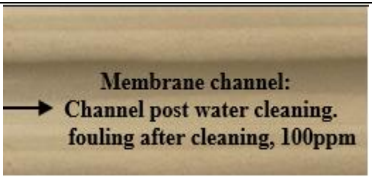
PFPT (1-1)	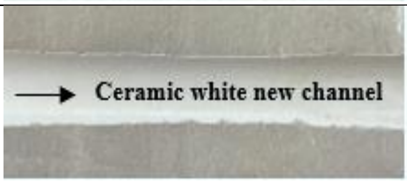	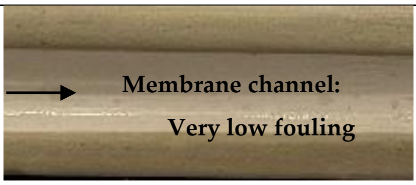	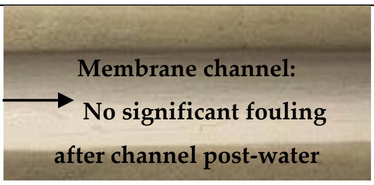
PFPT (1-2)	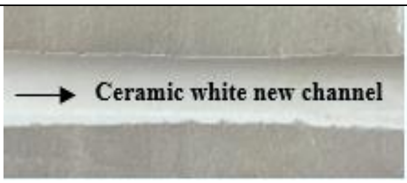	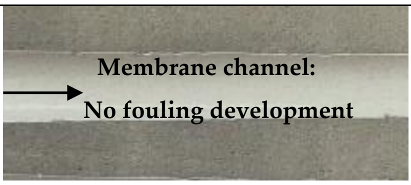	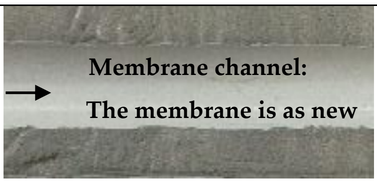
PFPT (2-2)	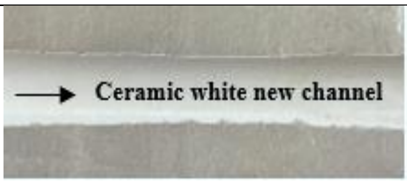	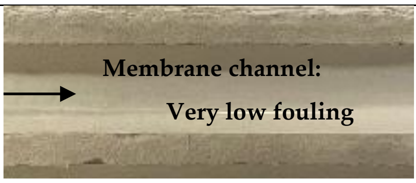	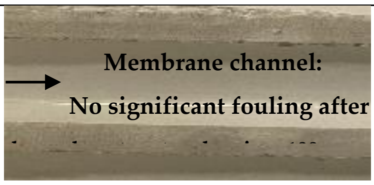

Figure S9. Ceramic membrane cross-sectional view before and after filtration and PFPT cycles, TMP=1.5 bar, CFV=1.0 m/s, and oil content of 100ppm.

	Internal channel ceramic membrane		
Feed 200ppm	At, t = 0 min<br>**new ceramic membrane is used for the experiment**	At, t = 120 min<br>**Fouled membrane At the end of the experiment**	**Water post-cleaned Ceramic membrane**
No PFPT	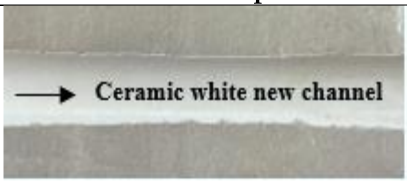	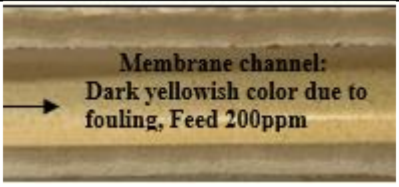	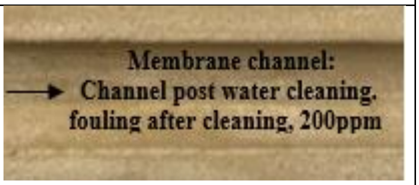
PFPT (1-1)	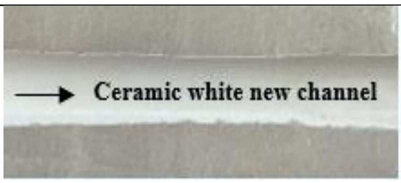	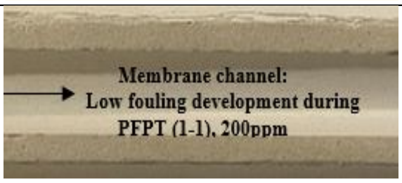	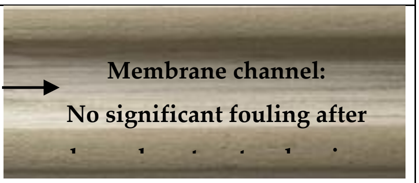
PFPT (1-2)	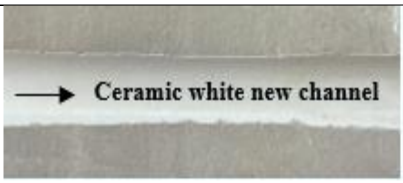	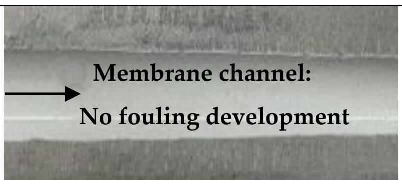	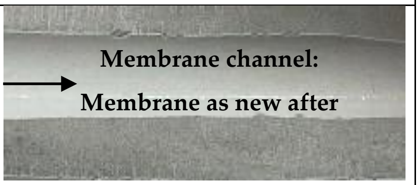
PFPT (2-2)	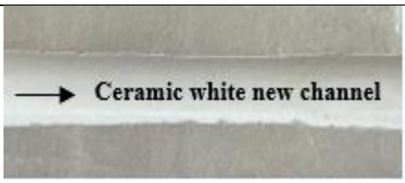	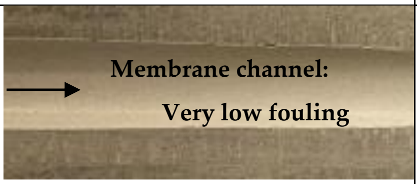	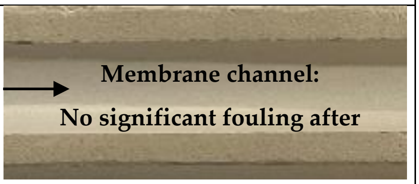

Figure S10. Ceramic membrane cross-sectional view before and after filtration and PFPT cycles, TMP=1.5 bar, CFV=1.0 m/s, and oil content of 200ppm.

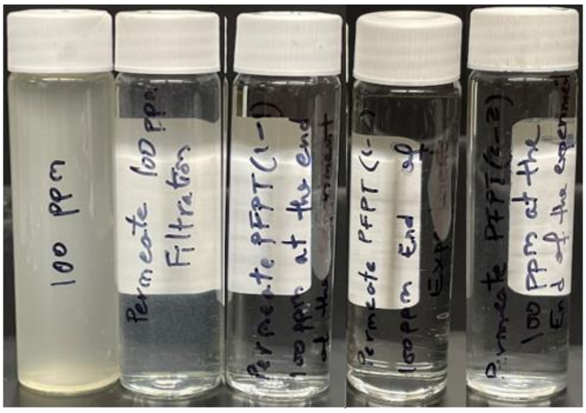

Figure S11. From left to right, feed 100ppm, no PFPT permeate, PFPT (1-1) permeate, PFPT (1-2) permeate, and PFPT (2-2) permeate.

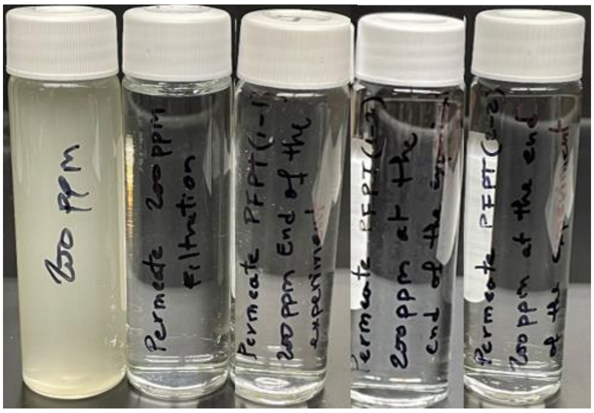

Figure S12. From left to right, feed 200ppm, no PFPT permeate, PFPT (1-1) permeate, PFPT (1-2) permeate, and PFPT (2-2) permeate.

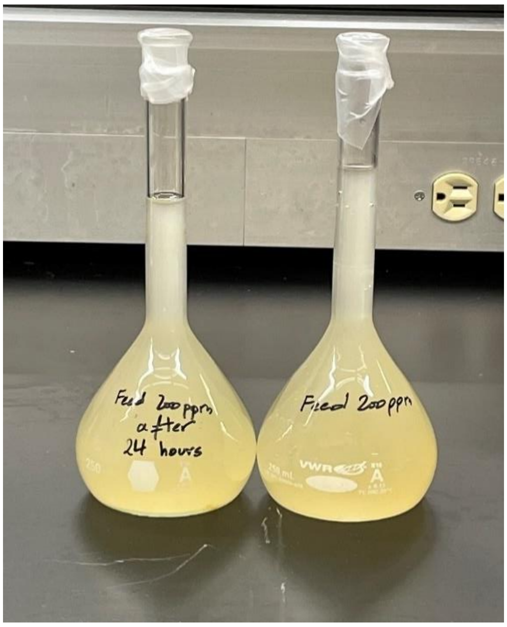

Figure S13. Oily wastewater emulsion (oil, water, and surfactant) at the synthesis time and after 24 hours: left volumetric flask shows no oil/water phase separation after 24 hours A16.

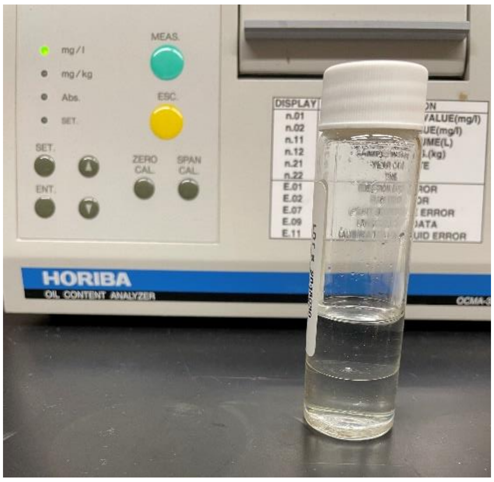

Figure S14. Oil phase extraction method, two-phase separation. Top layer: water. Bottom layer: solvent-316 and oil A17.

<table>
<tr><td></td><td colspan="3">Internal channel ceramic membrane</td></tr>
<tr><td>Normal Filtration (no PFPT)</td><td>At t = 0 min
new ceramic membrane is used for the experiment</td><td>At t = 120 min
Fouled membrane At the end of the experiment</td><td>Water post-cleaned Ceramic membrane</td></tr>
<tr><td>Feed 100ppm</td><td>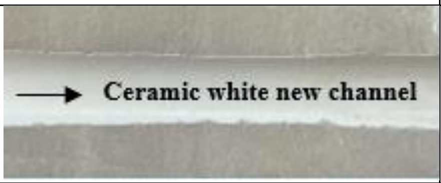
</td><td>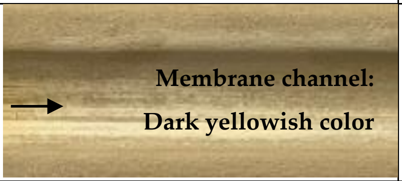
</td><td>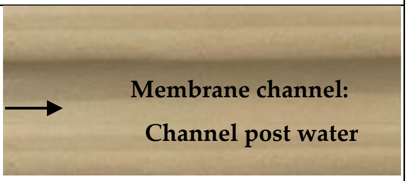
</td></tr>
<tr><td>Feed 200ppm</td><td>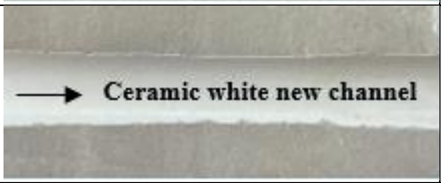
</td><td>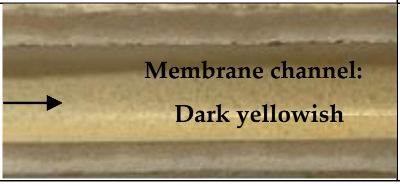
</td><td>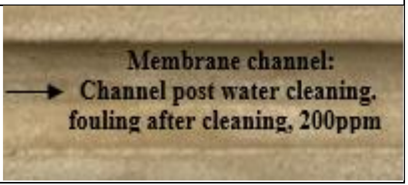
</td></tr>
</table>

Figure S15. Ceramic membrane internal channel surface oil deposition after normal filtration without PFPT cycles at TMP:1.5 bar, CFV: 1.0 m/s.

Table S1. The characteristics of the feed and permeate of the filtration and PFPT cycles at TMP=1.5 bar and CFV=1.0 m/s.

	Feed Oil content (ppm)	Permeate Oil content (ppm)	Rejection (%)
No PFPT (Normal filtration)	100	10.1	91
PFPT (1-1)	100	4.3	96
PFPT (1-2)	100	1.1	99
PFPT (2-2)	100	2.2	98

Table S2. The characteristics of the feed and permeate of the filtration and PFPT cycles at TMP=1.5 bar and CFV=1.0 m/s.

	Feed Oil content (ppm)	Permeate Oil content (ppm)	Rejection (%)
No PFPT (Normal filtration)	200	16.3	92
PFPT (1-1)	200	5.4	97
PFPT (1-2)	200	1.4	99
PFPT (2-2)	200	3.5	98

Table S3. The Turbidity of the feed 100ppm and permeate of the filtration and PFPT cycles at TMP=1.5 bar and CFV=1.0 m/s.

Feed 100ppm	Feed 100ppm Turbidity, NTU	Permeate Turbidity, NTU
No PFPT (Normal filtration)	430.9	1.65
PFPT (1-1)	430.9	0.53
PFPT (1-2)	430.9	0.25
PFPT (2-2)	430.9	0.36

Table S4. The Turbidity of the feed and permeate of the filtration and PFPT cycles at TMP=1.5 bar and CFV=1.0 m/s.

Feed 200ppm	Feed 200ppm Turbidity, NTU	Permeate Turbidity, NTU
No PFPT (Normal filtration)	562	2.49
PFPT (1-1)	562	1.55
PFPT (1-2)	562	0.44
PFPT (2-2)	562	0.63
